# Noninvasive assessment of autonomic function in human neonates born at the extremes of fetal growth spectrum

**DOI:** 10.14814/phy2.13682

**Published:** 2018-04-24

**Authors:** Hasthi U. Dissanayake, Rowena L. McMullan, Adrienne Gordon, Ian D. Caterson, David S. Celermajer, Melinda Phang, Camille Raynes‐Greenow, Michael R. Skilton, Jaimie W. Polson

**Affiliations:** ^1^ Boden Institute of Obesity Nutrition, Exercise & Eating Disorders The University of Sydney Sydney New South Wales Australia; ^2^ Sydney Medical School D17‐ Charles Perkins Centre The University of Sydney Sydney New South Wales Australia; ^3^ Royal Prince Alfred Hospital Sydney New South Wales Australia; ^4^ Sydney School of Public Health The University of Sydney Sydney New South Wales Australia; ^5^ School of Medical Sciences & Bosch Institute The University of Sydney Sydney New South Wales Australia

**Keywords:** Autonomic function, hypertension, in utero growth, newborn body fat

## Abstract

Birth weight is associated with adult cardiovascular disease, such that those at both ends of the spectrum are at increased risk. This may be driven in part by modification to autonomic control, a mechanistic contributor to hypertension. However, birth weight is a relatively crude surrogate of fetal growth; and newborn body composition may more accurately identify the “at risk” infant. Accordingly, we sought to determine whether newborns with high or low body fat have altered autonomic control of vasomotor function and cardiac contractility. Body fat was assessed by air‐displacement plethysmography <24 h postnatal. Measures of spontaneous baroreflex sensitivity (sBRS), blood pressure variability (BPV), and d*P*/d*t*_max_ variability were compared between newborns categorized according to established body fat percentiles: high body fat (HBF, >90th percentile, *n* = 7), low body fat (LBF, ≤10th percentile, *n* = 12), and normal body fat (control, >25th to ≤75th percentile, *n* = 23). BPV was similar across body fat percentiles; similarly, low frequency d*P*/d*t*_max_ variability was similar across body fat percentiles. sBRS was reduced in HBF compared to controls (11.0 ± 6.0 vs. 20.1 ± 9.4 msec/mmHg, *P* = 0.03), but LBF did not differ (18.4 ± 6.0 msec/mmHg, *P* = 0.80). Across the entire body fat spectrum (*n* = 62), there was a nonlinear association between newborn body fat and sBRS (*P* = 0.03) that was independent of birth weight (*P* = 0.04). Autonomic modulation of vasomotor function and cardiac contractility in the newborn did not differ by body fat, but newborns born with high body fat show depressed baroreflex sensitivity.

## Introduction

Hypertension is an important modifiable risk factor for cardiovascular and cerebrovascular diseases. Despite decades of research, over 95% of all cases of hypertension remain of unknown etiology (World Health Organisation, 2013), necessitating that treatments target the established symptoms and not the cause. A major consequence of this is a failure of the treatment strategies currently used in the management of hypertension with a considerable number of patients failing to adequately control their blood pressure (Yaxley and Thambar 2015).

A key pathophysiological hallmark of hypertension is elevated sympathetic nerve activity. Importantly, raised sympathetic activity has been reported in prehypertensive cohorts such as in patients with a family history of hypertension and may even be established during childhood (Julius et al. 1991). Thus, sympathetic overactivity precedes hypertension and may be a pathophysiological component of the causal pathway. Impaired baroreceptor reflex function has also been reported in both established hypertension (Bristow et al. 1969; Gribbin et al. 1971; Ducher et al. 2006), and in young adults with borderline hypertension (Takeshita et al. 1975; Matsukawa et al. 1991).

One of the most important recent advances in hypertension research is an understanding that hypertension often may have a developmental origin. This concept is based upon epidemiological studies where babies born small had a significantly higher prevalence of hypertension and cardiovascular disease in later life (Lawlor et al. 2002). People born small for gestational age represent a group at one end of the birth weight spectrum who are at increased risk of cardiovascular disease in adulthood, however, more recently those born large for gestational age also represents a group at increased risk (Koklu et al. 2007; Skilton et al. 2014).

Birth weight is unable to differentiate between the constitutionally small but well‐nourished newborn who has met their genetic growth potential, from an undernourished newborn of the same weight, whose intrauterine environment has restricted their growth trajectory. Similarly, those who are large for gestational age may be constitutionally large, or have excessive fetal growth above their expected growth trajectory. It has been proposed that groups of newborns with restricted or excessive growth in response to their intrauterine environment are those at most risk of later cardiovascular disease (Barker et al. 1993). Therefore, utilization of newborn body composition, comprising lean and fat mass may be a better indicator than birth weight.

Being able to measure autonomic function noninvasively and early detection of hypertension risk at a young age may help both in understanding the etiology of hypertension and identifying those at risk at a young age, which could prove highly valuable.

Accordingly, we sought to determine whether vascular autonomic function, specifically blood pressure variability and baroreflex function, differ by newborn percentage body fat and prematurity. Furthermore, this study has introduced a possible new noninvasive measure of autonomic regulation of the myocardium, the d*P*/d*t*_max_ variability in arterial pressure.

## Methods

### Ethical approval

This study was conducted in accordance with the standards set by the 2013 version of the *Declaration of Helsinki* and was approved by the Sydney Local Health District ethics committee and The University of Sydney Human Ethics committee (HRECH/14RPAH/478). Participation was voluntary, and informed written consent was collected from the parents of the newborn.

### Subject selection

Participants were recruited from the postnatal wards and the neonatal unit at Royal Prince Alfred Hospital, Sydney. Eligible newborns were singleton newborns between 37 and 42 completed weeks of gestation and those born late preterm between 34 and 36 weeks of gestation who had undergone routine body composition measurements. The only exclusion criterion for this study was major congenital abnormalities and ongoing need for respiratory support in the newborn. Obstetric assessment of pregnancies at risk of abnormal fetal growth (such as preeclampsia) was not excluded. Gestational age was calculated from first trimester ultrasounds.

### Body composition and anthropometry

Body composition was measured in infants in the first 24 h of life with air‐displacement plethysmography (PEA POD^®^, COSMED USA, Inc), as part of routine clinical practice. Air‐displacement plethysmography is an age‐appropriate method for assessing body composition (Fields et al. 2015), and has been validated in both term and preterm infants (Ma et al. 2004; Ellis et al. 2007; Roggero et al. 2012). This technique accurately measures body volume by the application of Boyle's law to the displacement of air by the infants in a sealed chamber. Fat mass and fat‐free mass are calculated by proprietary algorithms. Anthropometry was measured concurrently by two trained midwives. Weight is measured with the integrated PEA POD^®^ scales to the nearest gram, and head circumference to 0.1 cm. Length is measured with a length board to the nearest 0.1 cm (Easy‐Glide Bearing infantometer, Perspective Enterprises, USA). Newborns were categorized according to published body fat percentiles, adjusted for gestational age and gender (Hawkes et al. 2011).

### Data collection

Maternal and newborn characteristics were collected directly from mothers using a standardized questionnaire and corroborated from electronic medical records.

### Subjects

Blood pressure was recorded in 113 newborns, although the measurements from 43 of these were insufficient for analysis due to newborn‐ or equipment‐related problems. Failed measurements that were newborn‐related occurred when the newborn woke up during the measurement, failed to settle into sleep, or when the measurement was interrupted by a member of the clinical care team. Equipment‐related problems occurred when the blood pressure cuff failed to detect sufficient or continuous blood pressure. Adequate blood pressure waveforms were obtained in 70 newborns, of these 8 were late preterm newborns and 62 were full‐term newborns categorized according to established body fat percentiles (Hawkes et al. 2011) as follows: ≤10th percentile (*n* = male/female), *n* = 7/5; >10th to ≤25th, *n* = 5/5; >25th to ≤50th, *n* = 5/8; >50th to ≤75th^,^ *n* = 5/5; >75th to ≤90th^,^ *n* = 4/6; >90th percentile, *n* = 4/3.

To ensure equal spread across body fat percentiles for comparisons between the full‐term and late preterm group, the full‐term group was selected so that 10% of newborns were ≤10th body fat percentile, 15% from >10th to ≤25th, 25% from >25th to ≤50th, 25% from >50th to ≤75th, 15% from >75th to ≤90th and 10% of newborns >90th percentile body fat percentile. Individuals within body fat percentiles were chosen at random.

### Data acquisition

Continuous blood pressure recordings were acquired in the sleeping newborn at 1–5 days old using a noninvasive photoplethysomgraphic cuff (Finometer Pro, FMS, Finapress Medical Systems, The Netherlands), placed around the right wrist of the newborn with the sensor positioned over the radial artery. Blood pressure was recorded in 4‐minute intervals and repeated 1–3 times in each newborn. Analogue outputs of blood pressure were sampled at 500 Hz using Labchart program (ADInstruments, Sydney, Australia). Continuous blood pressure recordings were exported to Spike2 software (version 7.18, Cambridge Electronics Design, Cambridge, UK) and the following waveforms were generated: systolic blood pressure (SBP), d*P*/d*t*, and d*P*/d*t*_max_ of the blood pressure waveform.

### SBP variability

Power spectral analysis of the systolic blood pressure waveform provides a noninvasive method for the analysis of autonomic nervous system modulation to vasculature. SBPV was calculated using frequency domain methodology on the SBP waveform sampled at 5 Hz, with linear trend removal and by performing a Fast Fourier transform (FFT, 256 point, Hanning window, zero percent overlap) using customized algorithms in Spike2. The FFT was performed on SBP waveform durations of 1‐ to 2‐min epochs repeated 3–5 times and averaged in each infant, methodology similar to those previously published in infants (Yiallourou et al. 2012a, 2013). Spectral bands of SBPV were defined at 0.04–0.15 Hz for low frequency and 0.15–1.1 Hz for high frequency. The very low‐frequency band was not analyzed because of the short time period of the recordings. The high‐frequency band was based on respiratory rates in newborns at 0.5–1 Hz (Polson et al. 2006) and the total frequency band was defined as the range between 0 and 1.1 Hz.

The low‐frequency component of SBP variability is an established biomarker for sympathetic modulation of the vasculature. These low‐frequency oscillations of systolic blood pressure are a result from an oscillation of the sympathetic vasomotor tone and are enhanced during sympathetic activation (Julien 2006).

### Spectral analysis of d*P*/d*t*_max_ variability

**Figure 1:**
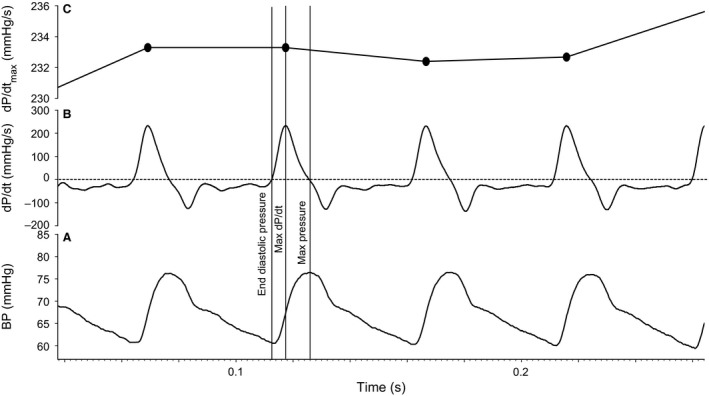
Example of blood pressure waveform (A) recorded in a control newborn using the Finapres and (B) d*P*/d*t* and (C) d*P*/d*t*_max_ derived from the BP waveform. The dP/dt waveform was generated by applying the slope function to the blood pressure waveform in Spike2. d*P*/d*t*_max_ coincides with the maximum upstroke of the blood pressure waveform during systole.

**Figure 2:**
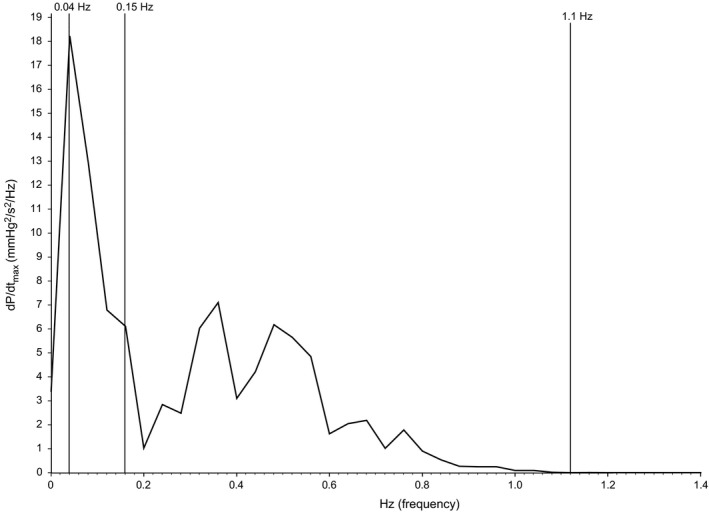
Estimated power spectral density obtained from a 60‐sec period of d*P*/d*t*_max_. The *y*‐axis represents d*P*/d*t*_max_ variability in mmHg^2^/sec^2^/Hz and the *x*‐axis represents frequency in Hz. The bin size was 0.04 Hz. The vertical lines denote the ranges for the very low‐frequency band (<0.04 Hz), the low‐frequency band (0.04–0.16 Hz), the high‐frequency band (0.16–1.1 Hz). Total power is the sum of the area under the curve between 0 and 1.1 Hz.

Estimated arterial d*P*/d*t*_max_ has been reported to be a surrogate measure for evaluating changes in left ventricular contractility (Rhodes et al. 1993), and d*P*/d*t*_max_ variability may represent autonomic modulation of ventricular myocardial contractility. The arterial pressure d*P*/d*t* is determined by the first differential of the blood pressure waveform, and d*P*/d*t*_max_, the peak rise in blood pressure was identified using a peak detection algorithm in Spike2 (Fig. 1). Frequency analysis of d*P*/d*t*_max_ variability was then performed similar to SBPV (Fig. 2). The d*P*/d*t*_max_ waveform was resampled at 5 Hz, and linear trend removal and FFT (256 point, Hanning window) was performed. As the maximum rate of change in arterial pressure is related to the force of ventricular contraction, the low‐ frequency component of d*P*/d*t*_max_ variability may reflect the sympathetic modulation of the myocardium during systole.

Measures of low and high power for SBPV and d*P*/d*t*_max_ were explored in its absolute values and using normalized units (nu) as some studies indicating power spectral components which are normalized for total power may better detect sympathetic and parasympathetic predominance (Pagani et al. 1997). Normalized units were calculated as previously described (Malik et al. 1996) using the equation below, as an example for normalized units of low frequency:

LF(NU)=(LF/(Total Power‐VLF))×100

### Baroreflex function analysis

Spontaneous baroreflex sensitivity (sBRS) was determined using the sequence method which incorporates the identification of sequences of consecutive increases in SBP (pressor ramps) or decreases in SBP (depressor ramps) that are followed by a progressive lengthening (or shortening) of pulse interval (PI) (Polson et al. 2006). Spontaneously occurring changes in SBP over a period of four or more beats were identified and the relationship with the corresponding pulse interval, with delays of three, four, and five beats were plotted. These delays were chosen based on a delay of approximately 2 sec for changes in heart rate in response to a change in blood pressure, given that the neonatal heart rate is ~2 beats per second (Polson et al. 2006). The slope and *r* ^*2*^ value of the linear regression for these plots were calculated and a baroreceptor mediated change in heart rate was only considered to have occurred when the slope was positive and *r* ^*2*^ was >0.8 for each delay.

In addition to sBRS, baroreflex effectiveness index (BEI) was used as an additional measure of baroreflex function in the newborn. This is a measure of barorflex recruitment and was determined as the ratio of the number of identified baroreflex sequences against the total number of SBP ramps observed for a given period of time (Rienzo et al. 2001), calculated using the formula below:

$$\text{BEI}=\frac{\text{Total number of pulse interval/SBP sequences}}{\text{total number of SBP ramps}}$$

### Statistical analysis

Statistical analysis was performed using SPSS Statistics, version 23 (IBM Corp, Armonk, N.Y., USA). Continuous data were expressed as mean (standard deviation), and categorical data as count (percentage). Data were visually inspected for normality, and nonnormally distributed data was transformed appropriately. One‐way analysis of variance (ANOVA), with Dunnett correction for multiple comparisons was applied to compare high body fat (HBF; >90th percentile) with control (body fat >25th to ≤75th percentile), and low body fat (LBF; ≤10th percentile) with control newborns. Full‐term and late preterm newborns were compared using independent‐samples *t*‐test. Chi‐square tests were used for categorical data. Nonlinear associations were determined by use of quadratic terms in multivariable regression models that included participants across the entire spectrum of body fat percent. Statistical significance was inferred where *P* < 0.05.

## Results

### Participant characteristics

Of the total 62 full‐term newborns, 7 were HBF (>90th body fat percentile), and 12 were LBF (≤10th body fat percentile) and 23 newborns in the control group (>25th to ≤75th body fat percentile). Mothers who gave birth to newborns with HBF had a greater proportion of cesarean deliveries and no labor compared to controls. Mothers in the LBF and control group were more likely to have a spontaneous, normal (vaginal) delivery, (Table 1).

**Table 1:** Maternal and newborn characteristics across body fat percentiles

	LBF ≤ 10th percentile (*n* = 12)	Control > 25th to ≤75th percentile (*n* = 23)	HBF > 90th percentile (*n* = 7)	*P* value
Maternal characteristics				
Age (years)	32 ± 4.4	34 ± 4.3	36 ± 6.6	0.26
Ethnicity, *n* (%)				
Asian	3 (25)	4 (17)	0 (0)	
Caucasian	4 (33)	15 (65)	5 (71)	
Middle Eastern	1 (8)	0 (0)	0 (0)	0.50
South Asian	3 (25)	3 (13)	1 (14)	
Other	1 (8)	1 (4)	1 (14)	
Maternal prepregnancy BMI (kg/m^2^)	21.9 ± 2.6	22.8 ± 3.5	22.8 ± 2.3	0.71
Gestational diabetes mellitus, *n* (%)	2 (17)	5 (22)	1 (14)	0.88
Preeclampsia, *n* (%)	1 (8)	0 (0)	0 (0)	0.28
NICU admissions, *n* (%)	1 (8)	2 (9)	0 (0)	0.71
Glucocorticoid exposure, *n* (%)	0 (0)	1 (4)	0 (0)	0.66
Maternal smoking, *n* (%)	0 (0)	0 (0)	0 (0)	–
Hypertension in pregnancy, *n* (%)	1 (8)	0 (0)	0 (0)	0.28
Mode of delivery, *n* (%)				
Normal delivery	10 (83)	13 (57)	2 (29)	
Instrumental	0 (0)	7 (30)	1 (14)	0.02
Cesarean	2 (17)	3 (13)	4 (57)	
Labor				
Spontaneous	6 (50)	14 (61)	2 (29)	
Induced	5 (35)	8 (35)	1 (14)	0.01
No labor	1 (8)	1 (4)	4 (57)	
Newborn characteristics				
Gestational age (weeks)	38 ± 1.0	39 ± 1.3	39 ± 1.1	0.18
Sex (girls/boys)	5/7	13/10	3/4	0.65
Birth weight (g)	2772 ± 332	3354 ± 469	4210 ± 315	<0.0001
Length (cm)	48 ± 1.8	50 ± 2.5	54 ± 1.7	<0.0001
Head circumference (cm)	33 ± 1.1	35 ± 1.5	36 ± 0.5	<0.0001
Body fatness (%)	3 ± 2	10 ± 2	17 ± 2	<0.0001
Body fatness (g)	98 ± 57	356 ± 101	728 ± 89	<0.0001
Fat‐free mass (%)	97 ± 1.8	90 ± 2	83 ± 5	<0.0001
Fat‐free mass (g)	2665 ± 302	3010 ± 400	3467 ± 299	<0.0001

Data are presented as mean ± SD for continuous data using one‐way analysis of variance (ANOVA), and *n* (%) for categorical data, using chi‐square tests between newborn body fat percentiles. LBF; low body fat percentile, HBF; high body fat percentile, NICU; neonatal intensive care unit.

Newborns with LBF were lighter, shorter, and had reduced head circumference compared to controls, while newborns with HBF were heavier, taller, and had a larger head circumference. As expected, based on selection, newborns with LBF had reduced body fat compared to controls, while newborns with HBF had higher body fat. Conversely, LBF newborns had increased fat‐free mass compared to controls, while newborns with HBF had reduced fat‐free mass, (Table 1).

Maternal characteristics were not different between the full‐term and late preterm group. Late preterm newborns were lighter, shorter, had reduced head circumference, body fat, and reduced fat‐free mass compared to full‐term newborns, (Table 2).

**Table 2:** Maternal and newborn characteristics of full‐term and preterm newborns

	Full term (*n* = 40)	Preterm (*n* = 8)	*P* value
Maternal characteristics			
Age (years)	33 ± 4.2	33 ± 5	0.66
Ethnicity, *n* (%)			
Asian	8 (21)	3 (38)	
Caucasian	23 (59)	4 (50)	
Middle Eastern	1 (3)	0 (0)	0.82
South Asian	5 (13)	1 (13)	
Other	2 (5)	0 (0)	
Maternal prepregnancy BMI (kg/m^2^)	22.9 ± 3.4	24.4 ± 9.2	0.67
Gestational diabetes mellitus, *n* (%)	6 (15)	2 (25)	0.49
Preeclampsia, *n* (%)	0 (0)	0 (0)	–
NICU admissions, *n* (%)	3 (8)	0 (0)	0.42
Glucocorticoid exposure, *n* (%)	1 (3)	1 (13)	0.20
Maternal smoking, *n* (%)	0 (0)	0 (0)	–
Hypertension in pregnancy, *n* (%)	0 (0)	0 (0)	–
Mode of delivery, *n* (%)			
Normal delivery	23 (58)	6 (75)	
Instrumental	9 (22)	1 (13)	0.65
Cesarean	8 (20)	1 (13)	
Labor			
Spontaneous	22 (55)	6 (75)	
Induced	12 (30)	1 (13)	0.54
No Labor	6 (15)	1 (13)	
Newborn characteristics			
Gestational age (weeks)	39 ± 1.1	36 ± 0.5	<0.0001
Sex (girls/boys)	23/17	3/5	0.30
Birth weight (g)	3387 ± 565	2737 ± 406	0.003
Length (cm)	50 ± 3	47 ± 2	0.012
Head circumference (cm)	35 ± 2	33 ± 2	0.04
Body fat (%)	11 ± 4	7.6 ± 2	0.05
Body fatness (g)	378 ± 183	204 ± 93	<0.01
Fat‐free mass (%)	89 ± 4	92 ± 2	0.05
Fat‐free mass (g)	3033 ± 423	2515 ± 324	<0.01

Data are presented as mean ± SD for continuous variables using independent *t*‐tests and *n* (%) for categorical variables, using chi‐square tests between full‐term and late preterm groups. NICU, neonatal intensive care unit.

### Autonomic function between newborn body fat percentiles

#### Systolic blood pressure variability

We found no differences in overall blood pressure variability or individual frequency components of blood pressure variability across body fat percentiles. Similarly, the normalized units of low and high frequency components were not different across body fat percentiles, (Table 3).

**Table 3:** Autonomic indices across body fat percentiles

	LBF ≤10th percentile (*n* = 12)	Control >25th to ≤75th percentile (*n* = 23)	HBF >90th percentile |(*n* = 7)	*P* value
Systolic blood pressure variability				
TP (mmHg^2^)	0.89 (2.98)	0.70 (1.50)	0.82 (1.29)	0.78
LF (mmHg^2^)	0.49 (1.60)	0.44 (0.95)	0.51 (0.77)	0.99
LF, NU	60.4 ± 16.5	68.5 ± 15.8	74.8 ± 12.6	0.12
HF (mmHg^2^)	0.25 (1.41)	0.21 (0.34)	0.12 (0.22)	0.58
HF, NU	39.6 ± 16.5	31.5 ± 14.8	25.2 ± 12.6	0.12
d*P*/d*t*_max_ variability				
d*P*/d*t*_max_ (mmHg/sec)	144.9 (91.94)	105.1 (69.6)	115.9 (45.2)	0.47
TP (mmHg^2^/sec^2^)	9.6 (27.92)	5.8 (19.7)	6.2 (11.0)	0.71
LF (mmHg^2^/sec^2^)	3.2 (6.3)	2.6 (4.5)	3.1 (3.0)	0.91
LF, NU	36.9 ± 15.8	38.3 ± 18.0	39.0 ± 16.3	0.96
HF (mmHg^2^/sec^2^)	5.3 (13.6)	3.1 (10.8)	3.1 (8.8)	0.78
HF, NU	63.1 ± 15.8	61.7 ± 18.0	61.0 ± 16.3	0.96
Baroreflex function				
sBRS (msec/mmHg)	18.4 ± 6.0	20.1 ± 9.4	11.0 ± 6.0	0.04
BEI	0.09 ± 0.05	0.15 ± 0.08	0.13 ± 0.08	0.09

Data presented as mean ± SD for normally distributed data and median (interquartile range) for log‐transformed data using one‐way analysis of variance (ANOVA). LBP; low body fat percentile, HBF; high body fat percentile, TP, total power; LF, low frequency; HF, high frequency; NU, normalized units; sBRS, spontaneous baroreflex sensitivity; BEI, baroreflex effectiveness index.

#### d*P*/d*t*_max_ variability

We found no differences in overall d*P*/d*t*_max_, d*P*/d*t*_max_ variability or individual frequency components of d*P*/d*t*_max_ variability across body fat percentiles. Similarly, the normalized units of low‐ and high‐frequency components were not different across body fat percentiles, (Table 3).

#### Baroreflex function

Spontaneous baroreflex sensitivity (sBRS) was significantly different between body fat percentiles (*P* = 0.04, Table 3). Multiple comparisons revealed that sBRS was ~45% lower in newborns with HBF than controls (*P* = 0.02), however, no differences were seen between LBF and controls.

Across the entire body fat spectrum, there was a nonlinear association between infant body fat percent and sBRS (*P* = 0.03, adjusted for gestational age and sex), which was independent of birth weight (*P* = 0.04 after adjustment). Body fat percent accounted for 13.4% of the variance in sBRS. In comparison, birth weight accounted for 10% of the variance in sBRS. In a model which included birth weight, the introduction of body fat percent accounted for an additional 7% of the variance in sBRS.

The baroreflex effectiveness was similar across body fat percentiles (*P* = 0.09), (Table 3).

### Autonomic function between late preterm and full‐term newborns

#### Systolic blood pressure and d*P*/d*t*_max_ variability

We found no differences in overall blood pressure variability, individual frequency components of blood pressure variability, or in any of the normalized units of low‐ and high‐frequency components between newborns born late preterm and those born full‐term, (Table 4).

**Table 4:** Frequency analysis of systolic blood pressure, d*P*/d*t*_max_ variability, and baroreflex function in newborns born full term or late preterm

	Late preterm (34–36 weeks) *n* = 8	Full term 37–42 weeks) *n* = 40	*P* value
Systolic blood pressure variability			
TP (mmHg^2^)	0.70 (1.99)	0.96 (1.67)	0.24
LF (mmHg^2^)	0.40 (0.63)	0.70 (1.17)	0.22
LF, NU	65.4 ± 19.0)	69.6 ± 15.0	0.50
HF (mmHg^2^)	0.21 (1.03)	0.23 (0.35)	0.46
HF, NU	34.6 ± 19.0	30.4 ± 15.0	0.50
d*P*/d*t*_max_ variability			
d*P*/d*t*_max_ (mmHg/sec)	115.4 (55.2)	120.6 (91.5)	0.94
TP (mmHg^2^/sec^2^)	6.23 (14.51)	5.55 (12.24)	0.80
LF (mmHg^2^/sec^2^)	2.27 (5.85)	2.87 (4.1)	0.75
LF, NU	44.7 ± 17.9	45.0 ± 15.7	0.99
HF (mmHg^2^/sec^2^)	2.7 (7.06)	3.00 (7.42)	0.78
HF, NU	55.3 ± 17.9	55.0 ± 15.7	0.99
Baroreflex function			
sBRS (msec/mmHg)	13.2 ± 7.1	18.0 ± 9.4	0.20
BEI	0.10 ± 0.05	0.2 ± 0.07	0.34

Data presented as mean ± SD for normally distributed data and median (interquartile range) for log‐transformed data, independent *t*‐test between preterm versus full‐term newborns. TP, total power; LF, low frequency; HF, high frequency; NU, normalized units; sBRS, spontaneous baroreflex sensitivity; BEI, baroreflex effectiveness index.

We found no differences in overall d*P*/d*t*_max,_ d*P*/d*t*_max_ variability or individual frequency components of d*P*/d*t*_max_ variability between newborns born late preterm and those born full‐term. Similarly, the normalized units of low‐ and high‐frequency components were not different between groups, (Table 4).

#### Baroreflex function

Spontaneous baroreflex sensitivity and baroreflex effectiveness index appeared to be reduced in late preterm newborns compared to full‐term newborns; however, this did not reach statistical significance, (Table 4).

## Discussion

There is strong epidemiological evidence demonstrating an inverse association between low birth weight and risk of cardiovascular disease in later life (Barker et al. 2005; Huxley et al. 2007). Recently, studies have also identified that being born large for gestational age may also impact cardio‐metabolic health (Eriksson et al. 2001; Koklu et al. 2007; Skilton et al. 2014). Being born preterm is associated with hypertension although studies have predominantly focused on severe prematurity, and it is unclear whether those born late preterm show similar cardiovascular maladaptation. One of the major identified modifiers of cardiovascular risk is altered autonomic function (Julius 1991; Parati and Esler 2012). We hypothesized that newborns with high or low body fat, or those born late preterm, may display changes in autonomic function that predispose them to cardiovascular disease, compared to term newborns with normal body fat. However, we found no evidence for altered autonomic modulation of vasomotor function and cardiac contractility at the extremes of the fetal growth spectrum or in newborns born late preterm. The exception was a reduced baroreflex sensitivity in newborns with high body fat, compared to those with normal body fat. This could be a factor that predisposes this group to development of hypertension in later life (Bristow et al. 1969).

### Baroreflex function and SBP variability in the newborn

In our study, newborns with HBF showed reduced sBRS compared to those with normal body fat. Studies in obese children and adults show a consistent reduction in baroreflex sensitivity (Skrapari et al. 2007; Lazarova et al. 2009; Calcaterra et al. 2013; Javorka et al. 2013), however, the time of onset of this change is unclear. Our results extend these findings by showing that reduced baroreflex sensitivity is also apparent in newborns with high body fat at just a few days postpartum. Decreased baroreflex sensitivity is a negative prognostic factor for cardiovascular morbidity and sudden cardiac death (La Rovere et al. 1998; Honzíková et al. 2006; Lazarova et al. 2009). Reduced sensitivity may be due to autonomic nervous system dysfunction (Spraul et al. 1994; Chapleau et al. 1995; Miller et al. 1999; Grassi et al. 2004) and or through changes in the mechanical properties of the arterial wall (Tanaka et al. 2001; Honzíková et al. 2006). Increased carotid intima‐media thickness and stiffness have been found in obese children (Woo et al. 2002; Iannuzzi et al. 2004; Skilton et al. 2014; Park et al. 2015), but there are currently no studies that have investigated the association of arterial stiffness with increased adiposity in the newborn. Reduced sBRS in this group may also be due to increased sympathetic activity as a result of increased plasma insulin or circulating leptin (Lazarova et al. 2009). Leptin has also been known to impair the cardiac baroreflex centrally at the level of the nucleus tractus solitarii (Arnold et al. 2009). We hypothesize that an imbalance in the autonomic nervous system, with an impaired parasympathetic activity alone or with sympathetic over activity may play an important role in the reduced sBRS observed in our newborns with high body fat. Interestingly, across our entire body fat spectrum, we found a nonlinear association between newborn body fat and sBRS, which was independent of birth weight. This may indicate that newborn body fatness may be a better predictor of spontaneous baroreflex sensitivity in the newborn than birth weight.

We found no differences in BPV between body fat groups, (Table 3). The magnitude of BPV in the low‐frequency band is regarded as an index of sympathetic modulation of the systemic vasculature and therefore total peripheral resistance (Pagani et al. 1986; Yiallourou et al. 2012a). The role of the high‐frequency component of BPV in autonomic regulation is less clear, however, a recent report indicated that it may be linked to respiratory modulation of sympathetic vasomotor tone (Menuet et al. 2017). Although we failed to identify any clear differences in BPV between groups, it is possible that differences may arise in later childhood. Currently there are no studies that have investigated BPV in the neonate or children with high adiposity. It therefore remains to be determined at what age autonomic dysfunction as observed through analysis of BPV at different levels of body fat may manifest.

Low birth weight is strongly, inversely associated with later cardiovascular disease (Barker et al. 2005), but does not discriminate between low birth weight due to fetal growth restriction or prematurity. In our study, newborns born full‐term with LBF showed no differences in baroreflex function or BPV. Studies in low birth weight children and adolescents born at term have shown increased BPV, measured in the time domain as the standard deviation from discontinuous noninvasive BP monitoring or standard deviation, coefficient of variation and deviation (Lurbe et al. 2001; Chen et al. 2012). These methodologies do not provide information on autonomic function as does frequency analysis of beat‐to‐beat BP waveforms and therefore cannot conclude whether the subjects of these studies had altered autonomic regulation. Because low birth weight is a strong predictor of later cardiovascular disease it is important to review whether changes seen in early life are due to prematurity or growth restriction alone, and at what age changes in autonomic function detrimental to cardiovascular risk may manifest. Future studies measuring BP waveforms in children and adolescents born small for gestational age and late preterm will help to answer this question.

### Baroreflex function and SBP variability in late preterm newborns

In our study, although preterm newborns showed a lower percentage body fat and low birth weight compared to their term born counterparts, no differences were observed in baroreflex or vasomotor function between these groups. Studies in newborns born preterm (28–32 gestational age) found reduced baroreflex sensitivity at 2, 3, and 6 months age (Witcombe et al. 2012). Studies by the same authors in children born preterm combined with fetal growth restriction did not affect BPV or baroreflex function; however, children born preterm alone showed increased high‐frequency BPV and no differences in baroreflex function (Cohen et al. 2017). It is unclear whether prematurity or growth restriction accounts for these changes seen in these studies. In our study, we did not look to separate fetal growth restriction within those born late preterm.

### Arterial d*P*/d*t*_max_ variability as an index of autonomic regulation of cardiac contractility

In our study, we found no differences in d*P*/d*t*_max_ variability across body fat percentiles or between full‐term and late preterm newborns. The d*P*/d*t*_max_ of left ventricular pressure is a well‐validated measure of contractility (Little 1985). Despite the d*P*/d*t*_max_ of the arterial pressure waveform being affected by a number of factors such as preload and arterial compliance (Adler et al. 1996), studies have shown good correlation between ventricular and arterial d*P*/d*t*_max_, and arterial d*P*/d*t*_max_ may offer a valuable methodology for noninvasive determination of myocardial contractility (De Hert et al. 2006; Masutani et al. 2009; Morimont et al. 2012).

A major influence on ventricular contractility is the autonomic nervous system (Charkoudian and Rabbitts 2009). This study has introduced a possible new measure, d*P*/d*t*_max_ variability as an index of autonomic control of myocardial contractility. We suggest that sympathetic modulation of the ventricular myocardium may be reflected in the low‐frequency component of d*P*/d*t*_max_ variability in a similar manner to how sympathetic modulation of heart rate and vascular resistance are reflected in the low‐frequency components of heart rate variability and BPV (Pagani et al. 1997; Yiallourou et al. 2012a). These factors are not independent, and both heart rate and blood pressure influence d*P*/d*t*_max_ because of their influence on preload and afterload. It is unlikely that the force‐frequency relationship, where myocardial contractility is observed to increase at higher heart rate, influences d*P*/d*t*_max_ variability because these effects are observed when heart rate increases over a sustained time frame (Janssen 2010), rather than with cyclical variations that are the hallmark of the d*P*/d*t*_max_ analysis. Moreover, the force‐frequency relationship appears to apply more to small mammals than human, at least under normal physiological conditions (Torres and Janssen 2011). Further investigation and validation of d*P*/d*t*_max_ variability independent of heart rate variability using specific inotropic and chronotropic agents is important and the subject of future studies. This added index of autonomic regulation of the myocardium may be important in evaluating pediatric and adult patients with autonomic dysfunction.

The use of radial (used in the newborn in our study) or finger arterial pressure derivative and its variability may provide valuable data to monitor cardiac performance noninvasively during daily activities. A limitation of this technique in the newborn is that it is unclear how accurate the radial arterial pressure derivative of d*P*/d*t*_max_ is in comparison to gold standard measures of cardiac contractility. However, as with the use of the Finapres in the newborn for determining BPV, beat‐to‐beat changes in d*P*/d*t*_max_ may be accurate even if the absolute measurement is not (Polson et al. 2006). It is the beat‐to‐beat change, rather than the absolute value that is important in the determination of d*P*/d*t*_max_ variability.

### Limitations

Obtaining blood pressure waveform in the newborn noninvasively using photoplethysmography is a technique that offers potentially important advances in both research and clinical settings (Andriessen et al. 2004; Polson et al. 2006; Yiallourou et al. 2012a, 2013; Cohen et al. 2017). However, the methodology is technically challenging, and often investigators are unable to obtain adequate blood pressure signals in subjects. Moreover, the accuracy of the measurements has been questioned (Drouin et al. 1997a,b; Andriessen et al. 2004; Polson et al. 2006). We have found previously that with appropriate cuff placement, there is good agreement between photoplethysmography and an arterial cannula in mean diastolic and systolic blood pressure measurements made over several minutes, however, on a beat‐to‐beat basis absolute measures of blood pressure were not well validated (Polson et al. 2006). Importantly, however, we found that measurement of the beat‐to‐beat changes in systolic blood pressure, and therefore calculations of systolic BPV and spontaneous BRS, were accurate (Polson et al. 2006).

This study is part of a wider study assessing early cardiovascular risk which included measures of heart rate variability obtain from the ECG, as another marker of autonomic control. However, due to the technical difficulties in measuring blood pressure waveform in the newborn, we were only able to obtain continuous blood pressure waveform data in a subset of participants. Thus, the sample size in the present report is reduced compared to heart rate variability measures. Given the large difference in the number of participants with available data, measures of heart rate variability were not included in this study and will be published elsewhere in conjunction with other markers of cardiovascular risk. The sample size in this study is, however, similar to, or higher than, other studies that have assessed autonomic function in the infant (Andriessen et al. 2004, 2005; Patural et al. 2004; Polson et al. 2006).

## Conclusions

There is strong evidence demonstrating that babies born at the extremities of the birth weight spectrum are at increased risk of developing cardiovascular disease in later life, although the mechanisms remain unclear. Moreover, the age at which predisposition for cardiovascular disease can be demonstrated is not known. In this study, we sought to ascertain whether babies born with high or low body fat, or those born late preterm, showed changes in autonomic function, compared to controls. We found little evidence for altered autonomic function of vasomotor function and cardiac contractility in these groups, with the exception of newborn with high body fat who showed reduced baroreflex sensitivity. Reduced baroreflex sensitivity observed in newborns with high body fat may be due to impaired parasympathetic activity or sympathetic over activity. Furthermore, across the entire body fat spectrum (*n* = 62), there was a nonlinear association between newborn body fat and baroreflex sensitivity which was independent of birth weight (*P* = 0.04). This study has introduced d*P*/d*t*_max_ variability as a possible new measure of autonomic control of myocardial contractility. This novel index, proposed as a measure of autonomic regulation of the myocardium, may be of value in evaluating pediatric and adult patients with autonomic dysfunction. Further investigation and validation of d*P*/d*t*_max_ variability using specific inotropic and chronotropic agents is important and the subject of future studies.

## Conflict of Interest

None.
